# Digoxin for the Management of Unstable Paroxysmal Supraventricular Tachycardia in a Patient Who Refused Electrical Cardioversion in a Rural Hospital

**DOI:** 10.1155/2023/7301460

**Published:** 2023-07-06

**Authors:** Belayneh Dessie Kassa, Mekbib Amede, Mollalign Wubante, Mebratu Libanos, Kumlachew Geta

**Affiliations:** ^1^Department of Emergency and Critical Care Medicine, Debre Tabor University, Debre Tabor, Ethiopia; ^2^Department of Internal Medicine, Debre Tabor University, Debre Tabor, Ethiopia; ^3^Department of Anesthesia, Debre Tabor University, Debre Tabor, Ethiopia

## Abstract

**Background:**

The most frequent atrioventricular tachycardia in the emergency room is atrioventricular nodal reentrant tachycardia (AVNRT). The first treatment option for ending stable narrow QRS complex SVTs is vagal maneuvers and adenosine. When adenosine or vagal maneuvers fail to change a patient's rhythm to normal sinus rhythm, long-acting AV nodal-blocking medications, including nondihydropyridine calcium channel blockers (verapamil and diltiazem), flecainide, or beta-blockers, are employed. Electricity (synchronized cardioversion) is the preferred form of treatment for unstable patients. *Case Presentation*. A 40-year-old male patient presented to the Emergency Department of Dubti General Hospital, the Afar regional state in Ethiopia, with a complaint of shortness of breath, palpitation, extreme fatigue, and chest pain of a day's duration. His blood pressure was 80/50 mmHg, he had cold extremities and a weak radial pulse, and his apical heart rate was fast, making it difficult to count. His electrocardiogram (ECG) showed paroxysmal supraventricular tachycardia (PSVT) with a heart rate of 200. He was a candidate for electrical cardioversion due to unstable PSVT, but he and his family members refused to give consent. Even though he is not indicated for pharmacologic therapy, none of the commonly used drugs were available at the hospital. We managed him with digoxin, and the outcome was positive.

**Conclusion:**

Even though we could not find a clear recommendation regarding the use of digoxin for patients with unstable PSVT (AVNRT), by taking into consideration its negative chronotropic effect and its action to suppress the AV nodal conduction velocity, it may reduce the heart rate, and it can be used as an alternative in such difficult scenarios and a resource-limited setting. But this should be further investigated.

## 1. Background

The Heart Rhythm Society estimates that millions of people will experience a heartbeat that is not normal at some point in their lives. They frequently occur in healthy individuals without heart diseases and are safe. Some heart rhythm problems, though, can be serious or even deadly. Arrhythmia risk can also be increased by underlying heart disease [1]. Fast beats that originate and are sustained in atrial or atrioventricular hub tissue over the bundle of His are referred to as paroxysmal supraventricular tachycardia (PSVT). Reentry phenomena and automaticity at or across the atrioventricular node are the causes of PSVT. Atrial tachycardia (AT), atrioventricular reciprocating tachycardia (AVRT), atrioventricular nodal reentrant tachycardia (AVNRT), and a few other tachyarrhythmias are all included in SVT [2]. The most frequent form of PSVT is AVNRT, followed by AVRT [3]. The type of rhythm that appears on the ECG and the patient's hemodynamic stability will determine how PSVT is treated in that patient. The ECG of patients who come with hypotension, shortness of breath, chest discomfort, shock, or altered mental status is evaluated to see if they are in sinus rhythm or not because they are deemed hemodynamically unstable. These people need urgent cardioversion if their heartbeat is not in sinus rhythm. If it is found that they are in acceptable sinus tachycardia, the underlying cause must be addressed [4]. Here, we present the case of a patient with unstable PSVT who refused electrical cardioversion at a resource-limited rural hospital in Ethiopia.

## 2. Case Presentation

M.D., a 40-year-old male patient, presented to the Emergency Department (ED) of Dubti General Hospital, the Afar regional state in Ethiopia, with a complaint of shortness of breath, palpitation, extreme fatigue, and chest pain of a day's duration. He had a dry, intermittent cough, a low-grade intermittent fever, and fatigue for a week before his current symptoms. He had no past medical or surgical history except that he was treated for pulmonary tuberculosis (PTB) six years ago. He denied alcohol intake, smoking, or any drug intake.

**Figure 1:**
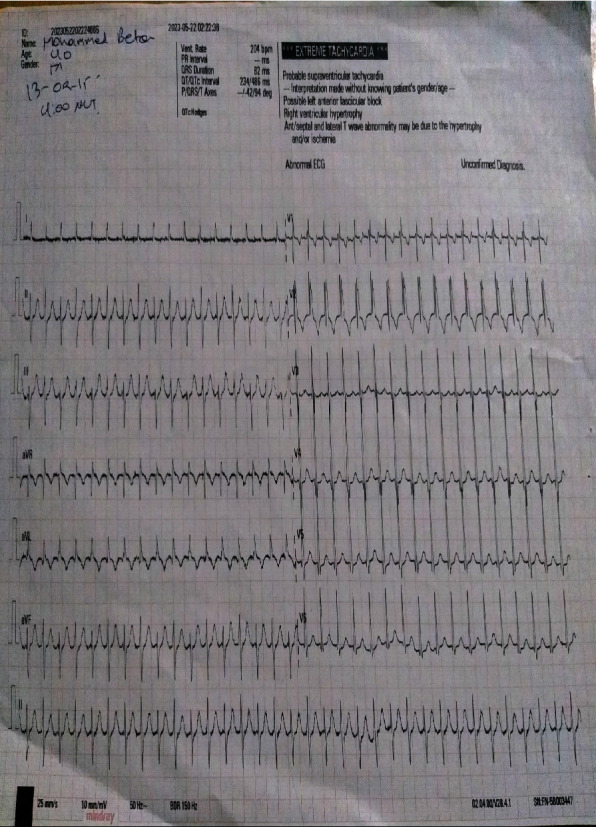
ECG before digoxin showing the AVNRT type of PSVT (regular rhythm, narrow QRS complex, no visible P-waves, pseudo-R-wave in V1, and HR of about 200).

On arrival, his blood pressure (BP) was 80/50 mmHg, his radial pulse rate was weak and fast, and his apical heart rate (HR) was also fast, making them difficult to count. His respiratory rate (RR) was 28/min, the temperature was 37.2°C, and his oxygen saturation was 98% with room air. He had cold extremities. Otherwise, he was fully conscious, and no other pertinent physical findings were noted. Immediately after arrival, he was transferred to the intensive care unit (ICU), and on a monitor, his HR was 208. A bedside ECG was done, which showed paroxysmal supraventricular tachycardia (PSVT) (Figure 1).

Considering his clinical presentations and ECG findings, unstable PSVT and pneumonia were entertained, and at this time, carotid sinus massage and modified Valsalva maneuvers were attempted, which proved ineffective. The patient and his family members were informed about the case scenario so that they could give written informed consent for synchronized electrical cardioversion. But the family members and the patient himself refused to sign informed consent due to fear and a bad perception of the outcome from the patient himself and due to religious influence from the family members.

**Figure 2:**
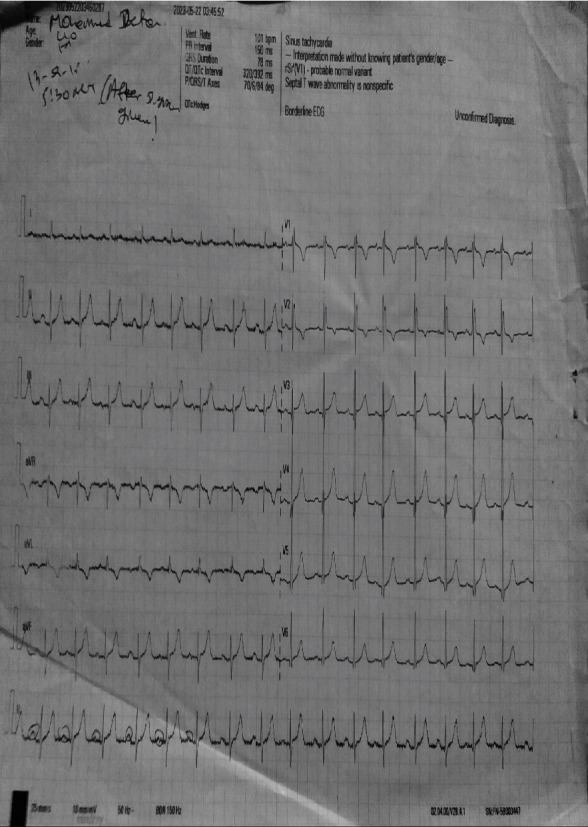
The ECG after digoxin was given showing sinus rhythm with an HR of about 100.

Even though he was not a candidate for pharmacotherapy because of instability, unfortunately, the commonly used drugs to manage PSVT (adenosine, beta-blockers, and calcium channel blockers) were not available in the hospital. This was because of the ongoing civil war in Ethiopia, which resulted in a shortage of foreign currencies to import essential drugs from developed countries and transportation problems from pharmaceutical supply agencies to hospitals. The only available drug was digoxin. Even though it is not a common practice to use digoxin to manage PSVT, taking into consideration its negative chronotropic effect and its action to suppress the AV nodal conduction velocity, digoxin 0.25 mg IV was given after informed consent was obtained from the patient and family members. After 10 minutes of digoxin administration, the HR dropped to 105 beats per minute, which were regular and full in volume. His BP came up to 95/65 mmHg, and his chest discomfort was relieved. At this time, a repeat ECG was done, which showed normal sinus rhythm (Figure 2).

He was investigated with a complete blood count (CBC), renal function test, liver function test, serum electrolyte test, and thyroid function test, but they were all normal. A chest X-ray and echocardiography were also done, and they turned out to be normal. He stayed in the ICU for 24 hours and was in stable condition. On the next day, metoprolol (12.5 mg PO BID) and amoxicillin/clavulanate (625 mg PO TID) were started, and he was transferred to the medical ward for observation. He stayed for 72 hours in the ward, and on his 4^th^ day of admission, he was referred to a better setup to have regular follow-ups with a cardiologist.

## 3. Discussion

The term “paroxysmal supraventricular tachycardia” (PSVT) refers to a class of arrhythmias with similar electrocardiographic characteristics but distinct causes that have been elucidated in recent years with the aid of sophisticated intracardiac recording and pacing techniques. The majority of cases are due to reentry, which is confined to the AV node, with sinus nodes, the atria themselves, and AV nodal bypass tracts causing Wolff-Parkinson-White syndrome. These types of supraventricular tachycardia are triggered by premature beats that dissociate the conduction of two pathways and allow the development of circulating electrical activity that extends to the atrial and ventricular myocardium [5].

The most frequent atrioventricular tachycardia in the emergency room (ED) is atrioventricular nodal reentrant tachycardia (AVNRT). A reentrant circuit involving the anterior and posterior inputs into the compact atrioventricular node is what causes AVNRT. AVNRT types are divided into two groups based on the ratio of atrial-His/His-atrial intervals: typical (slow-fast) and atypical (fast-slow and slow-slow). Another reentrant tachycardia is known as atrioventricular reciprocating tachycardia (AVRT), which necessitates the presence of an accessory route, a thin strand of myocardium that spans the typical insulation between the atria and ventricles [6].

Due to the atria and ventricles activating roughly at the same time in AVNRT, P-waves are difficult to detect on the electrocardiogram (ECG) strip. P-waves can be masked and either appear as a pseudo-S' deflection in inferior leads or as a pseudo-R-wave in lead V1. A more significant ECG finding with excellent diagnostic sensitivity is the pseudo-R-wave. An aVL notch, which appears as any positive deflection at the end of the Q-wave, R-wave, and S-wave (QRS) complex during tachycardia but is absent in regular sinus rhythm, is another ECG abnormality [7].

According to ACC/AHA/ESC guidelines, the first treatment option for ending stable narrow QRS complex SVTs is vagal maneuvers and adenosine [8]. Studies have demonstrated that adenosine can convert narrow QRS complex SVT to a normal sinus rhythm at a rate of 86.5% [9]. When adenosine or vagal maneuvers fail to change a patient's rhythm from narrow QRS complex SVT to normal sinus rhythm, long-acting AV nodal-blocking medications, including nondihydropyridine calcium channel blockers (verapamil and diltiazem), flecainide, or beta-blockers, are employed [10]. In investigations, diltiazem conversion rates ranged from 96 to 98.1% [11]. Flecainide was shown to have a 72% conversion rate to a normal sinus rhythm in AVRT patients and an 83% conversion rate in AVNRT patients [12].

Digoxin is useful in preventing AVNRT episodes in about 50% of instances and can be administered alone or in conjunction with beta-blockers. Digoxin treatment should be avoided in patients with accessory pathways and antidromic tachycardia or atrial fibrillation that are conducted via an accessory pathway because these medications may augment the conduction properties of the accessory pathway and cause an increase in ventricular rate or even ventricular fibrillation [13].

Adenosine is used more frequently because it is the safest and most effective medication in the acute context, but according to a review of the literature on the use of antiarrhythmic medications for the management of AVNRT in children older than 1 year, digoxin is still the medication of choice [14].

Electricity (synchronized cardioversion) is the preferred form of treatment for unstable patients. If the patient also exhibits chest discomfort, dyspnea, altered mental status, hypotension, pulmonary edema, or ischemic changes on the ECG, then all tachycardic rhythms, whether wide or narrow complex, are regarded as unstable. The suggested energy levels range from 50 to 200 joules for synchronized cardioversion. Starting with the least amount of energy (50 joules) and then doubling it if the shock is ineffective is the safest and simplest advice [15].

## 4. Conclusion

From an extensive literature review, we learned that the preferred management option for patients with unstable PSVT is synchronized electrical cardioversion. Even though we could not find a clear recommendation regarding the use of digoxin for patients with unstable PSVT (AVNRT), there are a few pieces of evidence to support its use in stable patients. But still, it needs further investigation to determine whether it can be used in an acute setting or not. This case represents the successful use of digoxin to save the life of a patient with unstable PSVT (AVNRT type).

Even though this is a single instance, reporting such cases is important for improving our knowledge regarding the options for the management of AVNRT, especially in such difficult scenarios in a resource-limited setting. Also, it might help medical professionals and researchers to do multicenter studies and experiments.

## Data Availability

This study was not supported by any data.
